# Effects of Healthcare Policies and Reforms at the Primary Level in China: From the Evidence of Shenzhen Primary Care Reforms from 2018 to 2019

**DOI:** 10.3390/ijerph19041945

**Published:** 2022-02-09

**Authors:** Mingyue Wen, Liao Liao, Yilin Wang, Xunzhi Zhou

**Affiliations:** 1 School of Politics and Public Administration, South China Normal University, Guangzhou 510631, China; mingyuewen@hotmail.com (M.W.); wylscu@hotmail.com (Y.W.); 2 Law School, Shanghai University of Finance and Economics, Shanghai 200433, China; zhouxunzhi2021@163.com

**Keywords:** primary care, health equity, policy formulation, policy implementation

## Abstract

Countries worldwide are making efforts to achieve health equity. China focuses on the implementation of the policy goal of “improving the primary level” to eliminate the health equity gap. The main purpose of this study is to examine the effects of the healthcare reforms at the primary level in China and to analyze the key factors that can help to improve their effectiveness. From the perspectives of the policy attention mechanism and public policy analysis, this study will explore primary care reforms from policy formulation to policy implementation on the basis of grounded theory and empirical research on primary care reforms in Shenzhen, China, that was conducted from 2018 to 2019. The present study found that the government pays close attention to the medical level and service level of primary care services at the policy formulation phase but less attention to talent level and information sharing. At the same time, this study combined with empirical data from primary care centers in Shenzhen for the period covering 2018 to 2019 evaluates policy implementation and its effect. Multiple regression analysis revealed that the medical level, talent level, service level, and information sharing helped to develop primary care services and improved health equity. Nevertheless, this study reflects a deviation between policy formulation and policy implementation for the development of primary care policies. Empirical experience shows that the development of talent level and information sharing can significantly promote primary care services and health management. Therefore, this study implies that in the process of promoting the health equity at the primary level, more attention should be paid to the consistency between policy formulation and policy implementation. Additionally, the policy promotion and influence mechanism can be improved, particularly in terms of talent development and information sharing, in order to effectively promote the development of health equity at the primary level.

## 1. Introduction

Reducing health inequality requires action across the whole of society. Although there is a great deal of scientific evidence on health inequalities, health policies require more evaluation, particularly in the countries where public health is fundamental in influencing the health level of society. China is a typical country that is dominated by a public medical system, and the healthcare resources vary from one region to another. On 2 March 2021, the China Health Commission national grassroots health work conference urged the Chinese government to thoroughly implement a healthcare policy “focusing on grass-roots units”. In China, eliminating health disparity has become a significant policy goal, particularly since the beginning of the COVID-19 pandemic. Against this background, primary healthcare development has been implemented as a strategy to achieve this target in China over the past ten years [1]. As important support for developing grassroots health units, national policy stipulates that, in principle, one community health service center should be set up within the jurisdiction of each sub district office or for every 30,000–100,000 residents. Although the national policy indicates the importance of equalizing primary healthcare services, their development is still unevenly distributed between China’s eastern and western regions. China’s healthcare resource agglomeration capacity had a gradual strengthening trend., with capacity weakening from east to west (the strongest in the east, the second strongest in central China, and the weakest in the west) [2]. Absolute inequalities in healthcare resources increased, while health utilization remained constant following China’s healthcare reform. Healthcare reform requires the continual recruitment of qualified healthcare workers and appropriately allocating healthcare infrastructures to strengthen the healthcare system’s capacity in impoverished areas [3]; in particular, the health problems that are experienced by vulnerable groups require government involvement. For example, given China’s aging population, the government should strive to improve the health of the elderly in China and to promote healthy aging by improving the elderly’s access to effective medical care and by encouraging their greater social engagement [4]. Although China has experienced several important stages of healthcare reform since the reform and opening up, the objectives and effects of each stage of reform have been limited by historical and social development. So far, China has mainly experienced three stages of healthcare reform at the national level.

In the first stage, before 2000, the promulgation of the Notice on the Implementation of Hierarchical Management of Hospitals (National Health Commission (Beijing, China) (89) No. 25), the Standard for Hierarchical Management of General Hospitals, and the Evaluation Measures for Medical Institutions (National Health Commission (Beijing, China) (1995) No. 30) extent balanced the allocation of regional medical and healthcare resources to a certain extent. This hierarchical management system gradually emerged and became better regulated. As such, the healthcare system has been built up at the comprehensive level, while the degree of medical coverage degree needs to be increased at this stage.

In the second stage, which took place from 2000 to 2009, three major medical insurance systems were successively established and gradually improved. In 1998, a State Council decision established a basic medical insurance system for urban employees that required that the system be promoted throughout the country, beginning in 1999. At the same time, the rural cooperative medical system began to be gradually reconstructed, and the medical insurance system for urban residents was established nationwide by 2009, thus forming a basic universal medical insurance system covering all people in China. Over the next ten years, China made substantial progress in improving equal access to healthcare and enhanced financial protection, especially for people of a lower socioeconomic status [5]. Therefore, the three medical insurance systems were successful in terms of improving the degree of medical coverage during this stage of reform. However, expensive medical treatments, the tension of medical resources, and the relationship between doctors and patients have been new problems since the second stage of reform.

In the third stage, which spanned from 2009 to 2014, new healthcare reform started to be implemented and aimed to reach the same level as that in moderately developed countries by 2020. “Ensuring national health” and “improving people’s health” became the strategic goal of this reform and in all healthcare policies. Thus, the primary care system has seen more pilot and implementation work since 2015. China’s new round of healthcare reform increased the coverage rate of basic medical insurance [6]. After the reform, the frequency of residents using inpatient services increased after the reform, and the averages of medical expenditures increased significantly, as did average medical expenditures, especially for uninsured and primary care services users. China also launched medical alliances (MAs) reforms to drive the development of primary medical institutions and to decrease health inequality in rural areas [7]. To enhance the pertinence, fairness, and accessibility of China’s medical and healthcare system, China has carried out a new round of healthcare reform that focuses on solving the problems of unbalanced development and difficult and expensive medical treatment, formulating policies from various angles to promote fair and accessible basic medical and healthcare services [8].

The main foci concentrate on the effectiveness of medical healthcare at this stage of reform and on promoting the primary care services to be realized for every resident in China, regardless of whether live in a city or in village. Although China has substantially introduced favorable policies for strengthening its primary care system, China still faces gaps and challenges, even after adjusting reform policies, aligning the practitioner and patient interests, or motivating social stakeholders [9,10,11,12], with governments and public policy still play a leading role in reforming these aspects. Although many studies have evaluated the healthcare policies in China from the perspectives of the supply of medical and healthcare resources and others have examined the main factors influencing the effects of the healthcare polices, little attention has been paid to the interactive process between the healthcare policy formulation and healthcare implementation and its effects. In particular, the perspective of the policy attention mechanism may help to provide a comprehensive view of this interactive process, as recent organizational theory and research has increased research regarding the determinants and consequences of attention in organizations that can be divided into the attentional perspective (top-down), attentional engagement (combining top-down and bottom-up executive attention), and attentional selection (the outcome of attentional processes) [13]. The theory of organizational attention distribution originated from Herbert A. Simon [14], and many classical theorists have selectively emphasized some aspects of attention distribution and structure while ignoring others [15,16]. Therefore, from the perspective of the policy attention mechanism, this study will try to provide new insights into healthcare policy formulation, implementation, and its deviations in order to examine the factors and processing details that affect healthcare policy.

Thus, this study explores how healthcare policies or reforms affect health management at the primary level in China and identifies the most important factors influencing health equity and management during the latest health reforms. Combining qualitative analysis and quantitative analysis, the study presents an overall analysis of recent healthcare reforms carried out from 2018 to 2019 in Shenzhen, China, to show the characteristics and the trends of the healthcare policies or reforms in China to form scientific policy implications.

## 2. Literature View

### 2.1. Evaluation of the Outcomes of China’s Healthcare Reform

China has been managing a fundamental transformation since the late 1970s. Focusing on a subset of China’s reforms, and since the 1990s, the healthcare system has shown that policy capacity is dynamic and adaptive in response to the changing challenges resulting from rapid transformation in the country [17]. So far, most studies have examined healthcare reforms in China from a policy-making process perspective by evaluating the outcomes of the reforms; some scholars point out that China’s healthcare policy has emphasized the interaction between the government and the grassroots levels in the healthcare field [18]. The public, as the demand side in the healthcare system, complain about access and distance to hospitals, the difficulties of being in queues for treatment, waiting times, expensive medical expenses, and so on [19]. Due to the government’s emphasis on the pursuit of policy value and healthcare form, it has responded to public opinion to a certain extent. On the other hand, public opinion has also strengthened the government’s political will and decision-making during healthcare reforms [20]. For example, in response to the public’s demand for equal health services, the Chinese government implemented the “new healthcare reform” in 2009, which led to an increase in the insurance coverage rate while creating a burden for low-income populations due to expensive healthcare services [21]. This leads to tension in the doctor–patient relationship. Other scholars have demonstrated that basic medical and healthcare services will undergo profound changes for both community healthcare service institution managers and medical staff after moving from the current model of medical and institutional development to one focused on community public relations [22]. Thus, the Chinese government needs to consider how to improve the doctor–patient relationship in the community as a policy goal in order to control the tension and to rebuild the doctor–patient relationship [23]. Moreover, using the internet as a source of health-related information helps to improve the doctor–patient relationship and to increase access to healthcare [24]. International scholars have evaluated government funding and government intervention in the development of internet medical care and government-led medical insurance initiatives. They suggest that the Chinese government adhere to its new healthcare reform policy and pay attention to demographic and economic factors when implementing it [25]. Others have found that China’s policy interventions strongly promote the development of internet hospitals in China. Political commitment, sustainable financial sources, and administrative capacity are strong driving factors for achieving universal health coverage (UHC) through health insurance reform [26].

From the perspective of policy effects, although many policies have promoted the diversified development of healthcare in recent years, the public’s sense of acquisition and satisfaction with the medical and healthcare supply has decreased. At the same time, although the supply of medical and healthcare resources has increased, the utilization efficiency has decreased [27]. For example, researchers measured the healthcare resource agglomeration capacities of 31 Chinese provinces (or municipalities) in 2004–2018 using the entropy weight method and found that policy recommendations to enhance the radiating role of healthcare resources in core provinces (or municipalities), rationally allocate health resources, and transform ideas to support public healthcare resource services were provided [28]. Healthcare resource distribution, poorly trained human resources, unguaranteed funding, and low patient acceptability were identified as implementation barriers [29].

### 2.2. Exploration of the Factors Affecting the Effectiveness of Healthcare Reform

Health is an important social policy issue that is influenced by health policies and social policies [30]. It would be important to explore and improve the healthcare system from the perspective of social policy process and analysis [31]. In many middle-income countries with healthcare systems that are under reform, difficult social problems and challenges are coming to the forefront. For example, Poland has recently intensified its health promotion to extend its healthy life expectancy and to reduce health inequalities. Moreover, geographic distribution, inequalities in wealth distribution, and education are likely to significantly affect the achievement of the malnutrition targets that have been set in Bangladesh. The government of Bangladesh has prioritized and implemented interventions in order to reduce inequalities [32]. In India, substantial inequality exists at the regional, geographic, economic, and social levels, and various socioeconomic factors lead healthcare inequalities. However, through a noticeable improvement in maternal healthcare services, the maternal mortality ratio has significantly declined in India [33]. Meanwhile, other research has demonstrated that the number of doctors and therapists, number of support clinics for home healthcare facilities and home-visit treatments, and dentistry expenditure per capita were positively correlated with life expectancy and healthy life expectancy [34]. There was inequality in healthcare-related human resource distribution in mainland China in 2014. However, the economic status of the cities on the mainland were the main cause of this inequality [35].

At the same time, other researchers examined the introductions of intervention policies, showing that the policy focused on the improvement of socioeconomic status, effectively reducing health disparities [36]. Compared to urban residents, rural residents experienced greater levels inequality in terms of the demand and utilization of healthcare services as well as in terms of annual health and hospitalization expenditures [37]. Thus, the development of health equality depends on local socioeconomic status.

Although many studies have evaluated healthcare policies in China from the perspectives of the supply of medical and healthcare resources, the utilization efficiency, or the resource allocation degree at the regional level and some other studies have examined the main factors influencing the effects of the healthcare policies, such as the number of doctors and therapists, support clinics, and home-visit treatments, little attention has been paid to the interactive process between healthcare policy formulation and implementation and its effects. Thus, policy makers and health care workers might have a lack of comprehensive understanding of the problems associated with healthcare policies or reforms. This study aims to obtain a more accurate understanding and more comprehensive analysis of the dynamic process of the healthcare reforms at the primary care level, which may provide necessary insight for increasing the effectiveness of healthcare policies.

## 3. Research Backgrounds and Research Design

### 3.1. Research Backgrounds

In February 2010, the State Council of China declared that all provinces (autonomous regions and municipalities) should choose one to two cities or urban areas to carry out the pilot reform for public hospitals. Shenzhen was selected as one of the first national contact pilot cities. Since 2010, to ensure that 10,000–20,000 people in each community in Shenzhen are able to receive primary care center services, around 700 primary care centers have been built and developed. At the same time, these primary care centers must be registered with detailed resident health records. Registered residents make up 60% of the registration rate, and 20% includes temporary residents. Meanwhile, in 2017, the Shenzhen Municipal Health Commission designated the year of 2017 as the “year for improving the quality of healthcare services at the primary level” and mainly promoted the development of primary care services. Therefore, an in-depth study of the development of primary care in Shenzhen is conducive to outlining the degree of expected challenges for the current development of primary care and for evaluating the effect of the healthcare reforms and policies. Moreover, this research will not only examine policy formulation, but it will also evaluate the effect of primary care (based on the updated practice data) under the reform to facilitate a more accurate understanding and more comprehensive analysis of the dynamic process of primary care services and the characteristics of different types of primary care centers, later summarizing the main healthcare reform trends at the primary level.

### 3.2. Research Framework and Hypothesis

This study employed qualitative and quantitative methods to conduct an overall analysis of the recent healthcare reforms. The study mainly examines the effects of healthcare reforms at the primary level and analyzes the key factors that may help to improve their effectiveness from the perspectives of the policy attention mechanism and public policy analysis on the basis of grounded theory and the previously conducted empirical research on primary care reforms in Shenzhen, China, from 2018 to 2019 since in 2017, the Shenzhen Municipal Health Commission designated 2017 as the “year for improving the quality of healthcare services at the primary level”, with the majority of the promotion for the development of primary healthcare services taking place since 2017 and has mainly promoted the development of primary care services since 2017.

As NVivo (NVivo is the premier software for qualitative data analysis. Qualitative researchers use it to describe, evaluate, and interpret social phenomena. They analyze data from interviews, surveys, field notes, web pages, and journal articles and work in sectors ranging from social science and education to healthcare and business. NVivo allows researchers to organize, analyze, and visualize their data to find the patterns it contains. https://www.qsrinternational.com/NVivo-qualitative-data-analysis-software/home, accessed on 20 January 2022) can analyze word frequency in documents, this study assumes that the words that appear in policy documents with a high degree of frequency can reflect the attentional perspective, attentional engagement, and attentional selection of policy makers during the policy formulation phase. This study conducted NVivo coding analysis on official healthcare documents outlining policies and reforms at the national, provincial, and city levels of Shenzhen from 2018 to 2019 to reveal the distribution of policy attention during the policy-making phase and then used Stata 15.0 (Stata 15.0 is manufactured by StataCorp LLC and sourced in Guangzhou, China.) to conduct multiple regression analysis to examine empirical evidence related to the policy implementation by the practitioners and patients at primary care centers. Per grounded theory, three levels of coding: open coding, axial coding, and selective coding, are required when conducting NVivo coding analysis.

Stage 1—open coding. This is defined as “the analytical process through which the concepts are identified and the properties and dimensions of the data are discovered”.

Stage 2—axial coding. This procedure is defined as a thorough category analysis and aims to identify the interactions and relationships between such a category and other categories/subcategories or properties.

Stage 3—selective or theoretical coding. Theoretical codes “weave the fractured story back together again”.

Stage 4—theoretical saturation. Data gathering is terminated, as no more new ideas or relationships appear [38].

The proceeding NVivo results are shown in Table 1 and Table 2 as follows:

According to the analysis results regarding the healthcare policies and reforms at the national, provincial, and city levels of Shenzhen from 2018 to 2019, this study can summarize five important dimensions shown in Table 3 within these reforms that reflect the attention of the policy makers: primary care service effects, medical level, talent level, service level, and information sharing. As all of these factors are important for exploring the development of primary care services. Moreover, based on previous studies, primary care service effects are influenced by medical level, talent level, service level, and information sharing. Thus, this study will develop a research design to explore empirical evidence from the policy attention mechanism accordingly in order to examine how the medical level, talent level, service level, and information sharing will affect primary care services during the policy implementation phase. The study examined the key factors, evaluated whether and how healthcare policies have significantly affected health equity at the primary level, and developed the following research hypotheses:

The choice of patients selecting primary care centers is mainly affected by the quality of medical services provided by primary care centers and the service difference compared to other medical institutions [39]. Patients will only choose primary care centers if the quality of the medical services provided by these centers can be improved, allowing patients to receive satisfactory medical treatment [40]. Thus, the Hypothesis 1 is:

**Hypothesis** **1.** *The higher the medical level is, the higher the primary care service effects are.*

Moreover, reduced waiting lists and increased admission rates as well as the professional competences of primary care doctors can increase satisfaction from the perspective of both healthcare service providers and patients and their families [41]. Thus, Hypothesis 2 is:

**Hypothesis** **2.** *The more talent training is developed, the higher the primary care service effects are.*

It is well known that primary healthcare resources greatly affect service accessibility and resident willingness to visit primary healthcare centers [42]. Additionally, the financial stability of financial care centers is related to their capacity to purchase basic equipment, which affects service benefits and effects [43] [44]. Following this, Hypothesis 3 is:

**Hypothesis** **3.** *The higher the service levels are, the higher the primary care service effects are.*

Finally, with the rapid development of information technology and the implementation of China’s health strategy, an integrated healthcare system with primary care centers will provide convenient and accurate medical services for community residents and the elderly [45]. As such, the Hypothesis 4 is:

**Hypothesis** **4.** *The higher the information sharing level is, the higher the primary care service effects are.*

### 3.3. Data Collection and Collation

The data in this study were drawn from the panel data from 174 primary care centers in Shenzhen from 2018 to 2019 (including 139 in the Bao’an District and 35 in Pingshan District). First, the Bao’an District, which is located in the western part of Shenzhen City, is an economic and populated district. In 1996, the first primary care center in Guangdong Province was developed in the Bao’an District. The Bao’an District has an area of 397 square kilometers, accounting for 19.9% of the city of Shenzhen, and had a GDP of CNY 361.2 billion in 2018. The district has a local population of 4,476,554 people. In 2018, there were 139 primary care centers in the Bao’an District, which passed the national evaluation, allowing the district to become one of the first pilot areas for national primary care centers. In the intervening years, the Bao’an District has encountered almost all of conceivable problems associated with developing a primary care center. Retaining patients is the biggest problem facing the Bao’an District. There are only two high-quality medical hospitals in the Bao’an District. Its primary care centers are relatively developed for Shenzhen, concentrating medical and healthcare resources and forming a basic medical service mode based on public social health centers. In the Bao’an District, healthcare policy has paid attention to many details to improve primary care service levels. For example, it requires hospital experts to be stationed in the primary care center for no less than one year, has adjusted performance assessment standards, and has improved minimum standards for outpatient subsidies and other measures.

On the contrary, the Pingshan District, which is outside Shenzhen city center, is adjacent to the city of Huizhou, and is home to a variety of electronic plants with large numbers of workers. There is no high-quality hospital in the Pingshan District, only a secondary hospital that is relatively remote. The Pingshan District has an area of 168 square kilometers, accounting for 8.4% of the city of Shenzhen and had a GDP of CNY 80.11 billion in 2018. The district has a local population of 551,333 people. In 2018, there were 35 primary care centers in the Pingshan District, demonstrating a trend of rapid development. Nevertheless, private primary centers represent the main healthcare trend in the Pingshan District. Primary care centers are a relatively recent development in the Pingshan District and have relatively poor medical and healthcare resources, forming a basic medical service mode that mainly based on private primary centers. The Shenzhen Municipal Health Commission has formulated several policies in response to this health equity issue, such as standardizing the management measures for basic public health services, standardizing integrated medical and nursing services, providing financial subsidies for basic medical services, and ensuring the public welfare of the primary care center.

Because the individual indicators selected in this study could not be obtained due to missing records, the missing values in the database were supplemented with relevant data with high reliability, including interview data. The collected data include dependent variables—primary care service effects, independent variables—medical resource and talent training, and control variables—information sharing and service level. This research systematically integrated these aspects to analyze the characteristics and differences in the development of primary care services based on the diversified medical mode formed in different districts in order to understand the dynamic process of primary care services and to summarize the characteristics of different development types of primary care centers.

In the primary care service effects dimension, this study selected three health effect level indicators: “annual number of hypertension patients”, “annual number of diabetes patients,” and “annual number of the elderly.” According to the National Code for Basic Public Health Services, these three indicators reflect the degree of standardization in primary care services and, to a certain extent, how healthy the community residents are.

The medical level adopted “number of general practitioners”, and the talent level adopted “number of deputy chief physicians” as measurements to investigate professional level of the primary care centers, as the number of doctors represents different medical service capacities.

In the service levels dimension, the study selected “annual outpatient expenses” and “annual total medical income (including financial appropriation)” to measure the patient expenditures and the financial income of the primary care centers.

In the information sharing dimension, this study selected one commonly used efficiency performance indicator (“annual two-way referral number”) to measure the output and input effects of the service provided by the primary care center. Public primary care centers are predominant in the Bao’an District, while private primary healthcare centers are predominant in the Pingshan District. Therefore, both public and private primary healthcare centers were included. The variables set for this study are shown in Table 4.

## 4. Analysis Results

### 4.1. Characteristics of the Development of Shenzhen Primary Care Center

Through data transformation and collation, Table 5 reports a total description of the results of the statistical analysis of the relevant factors related to this study. First, the medical and talent level of the primary care service centers is also called the construction of the medical and health personnel team. In general, the allocation of doctor resources in primary care centers is not reasonable. Specifically, the number of GPs in primary care centers vary widely, with the largest number being 44, while some communities have none. There are fewer than three doctors in each community on average, and the total number of doctors is not large.

The service levels of the primary care centers differ widely. The average outpatient expenditure for primary healthcare center was determined to be CNY 407,174.30, with the average total medical income being CNY 50,384.45. Some primary care centers have no annual income, while some primary healthcare centers earn CNY 99,197 each year.

From the perspective of information sharing, the maximum number of two-way referrals in the different primary care centers was over 40,000, more than ten times the average.

In terms of the effects of primary care services, each primary care center regularly manages an average of 95 patients with diabetes, 218 elderly people, and more than 290 patients with hypertension. Moreover, the maximum number of these three groups was about five times the average number at the standardized management level.

### 4.2. Comparative Analysis of the Development of the Two Types of Primary Care Centers in Shenzhen

Table 6 presents a description and the statistical analysis results of the primary care centers in the two districts and reveals that there are lots of differences in terms of the development of the primary care centers between the two districts and two types of centers (public primary center and private care center) in the different socio-economic contexts.

In terms of medical and talent level, there was a gap between the doctor resources in the Pingshan District and Bao’an District, and the overall number of doctors was not large. The centers in the Bao’an District had a maximum of 44 GPs and an average of 13 GPs; the Bao’an District had a maximum of 40 GPs and an average of 11 GPs. In contrast, the maximum number of deputy chief physicians in the Bao’an District (15) was higher than the number of deputy chief physicians in Pingshan (8), while the average number of deputy chief physicians in primary healthcare centers was 2.80 in Bao’an and 1.43 in Pingshan.

In terms of service levels, the average outpatient expenses in the Pingshan District were CNY 285,159.70 compared to CNY 326,347.40 in the Bao’an District. Additionally, the maximum amount of outpatient expenses in the Pingshan District were CNY 983,300, compared to CNY 974,599 in the Bao’an District. The average total medical income was CNY 357,312.30 in the Pingshan District and CNY 419,501.80 in the Bao’an District, while the maximum total medical income was CNY 989,180 in Pingshan and CNY 991,972 in the Bao’an District. In general, the outpatient expenses and total medical income levels were generally higher in Pingshan than they were in Bao’an, while the average levels were generally lower.

In terms of information sharing, the number of two-way referrals was quite different in the two districts, with the centers in Bao’an District having more than their counterparts in the Pingshan District. The maximum number of two-way referrals in the Bao’an District was 44,310, compared to 14,255 in the Pingshan District. Generally, centers in the Pingshan District had fewer patients than those in the Bao’an District.

In terms of primary care service effects, the centers in the Bao’an District managed more total patients than the centers in the Pingshan District as well as more elderly patients and patients with hypertension or diabetes. The mean values for centers managing patients with hypertension, diabetes, and elderly patients were 322.56, 105.23 and 242.78, respectively, in the Bao’an District compared to 157.26, 49.54, and 115.72 in the Pingshan District.

### 4.3. Correlation Analysis

To test the impact of healthcare policy on the primary care service effects, the stepwise regression analysis method was used, and three models devised. The explanatory variable of Model 1 was the total number of GP practitioners in the primary care centers; Model 2’ s explanatory variable was the number of deputy chief physicians at the primary care centers; the explanatory variable of Model 3 was the total number of GP practitioners and the number of deputy chief physicians in the primary care centers. The control variables for all three models were outpatient costs and total hospital revenue (for service levels), the number of two-way referrals (information sharing), and hospital type.

Table 7 reports the correlation coefficients for the three primary care service effects models, all of which were less than 0.7, indicating no multiple collinearities between the variables of the three models; thus, no explanatory variables were removed.

### 4.4. Results of the Model 1–Model 3 Regression Analysis

**Figure 1:**
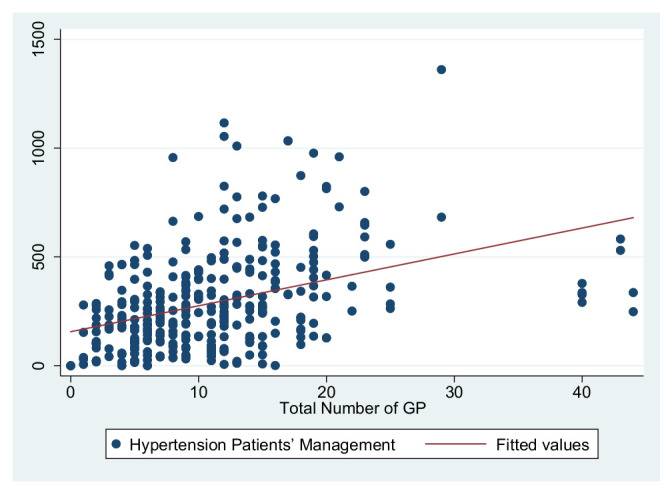
Scatter diagram of hypertension patient management and total number of GPs.

**Figure 2:**
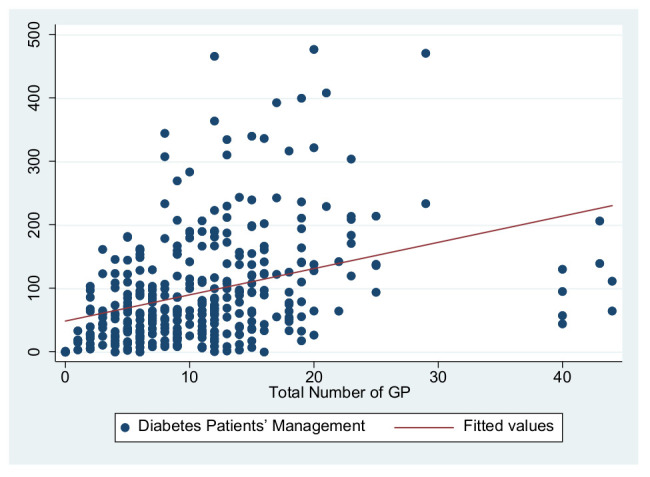
Scatter diagram of diabetes patient management and total number of GPs.

**Figure 3:**
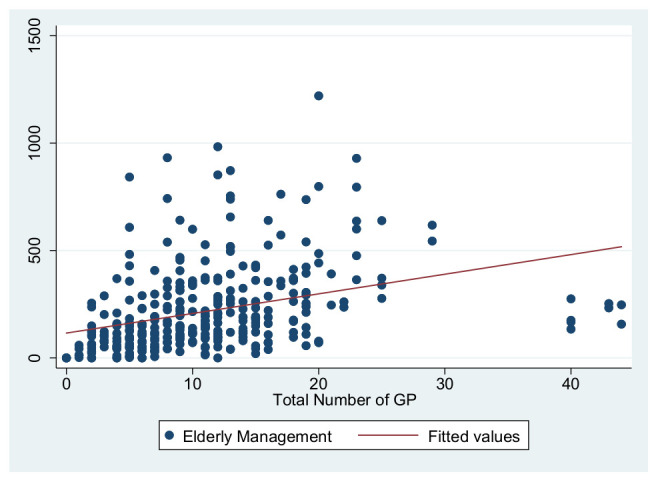
Scatter diagram of elderly management and total number of GPs.

**Figure 4:**
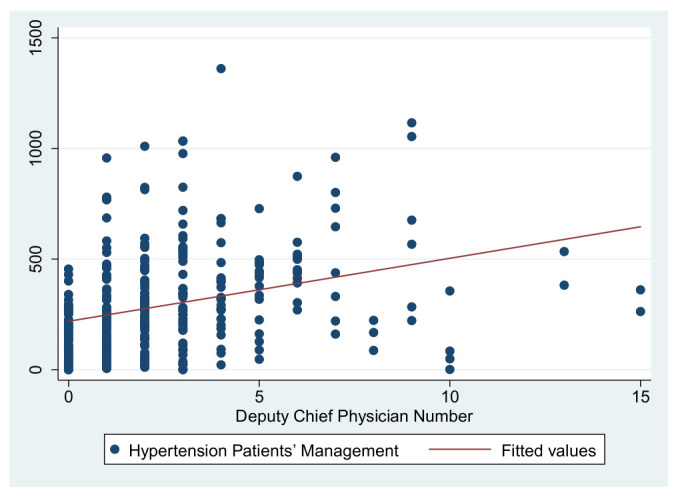
Scatter diagram of hypertension patient management and total number of deputy chief physicians.

**Figure 5:**
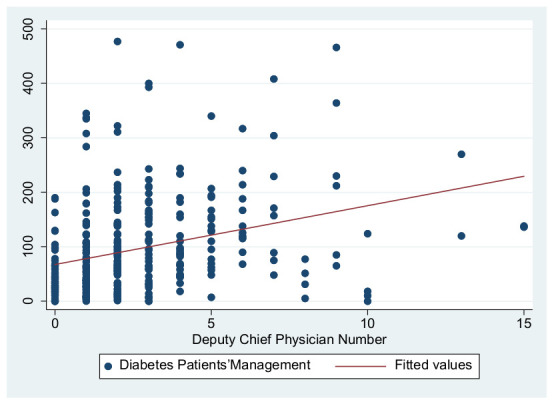
Scatter diagram of diabetes patient management and total number of deputy chief physicians.

**Figure 6:**
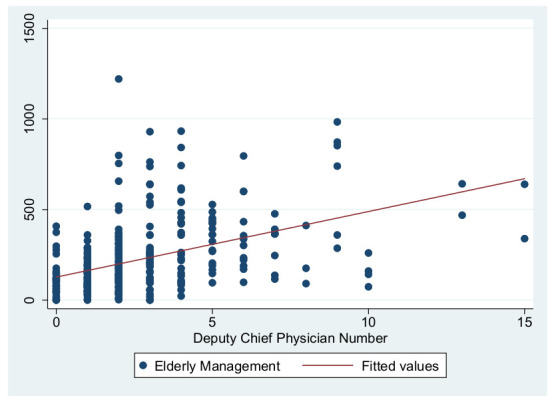
Scatter diagram of elderly management and number of deputy chief physicians.

As previously shown in Table 4, the dependent variables in this study are the primary care service effects, the independent variables are the total number of GPs to represent the medical level and the number of deputy chief physicians to represent the talent level, and the control variables are service levels and information sharing. These were used to construct three primary service effects models. The independent variable in Model 1 is the total number of GPs, the independent variable in Model 2 is the number of deputy chief physicians, and the independent variables in Model 3 are the number of GPs and the number of deputy chief physicians. Figure 1, Figure 2, Figure 3, Figure 4, Figure 5 and Figure 6 show the scatter diagrams of the dependent variables management of patient with hypertension, management of diabetes PA ties, and elderly management and the independent variables total number of GPs and the number of department chief physicians, all of which present a positive correlation.

Table 8 reports the regression analysis results for the three primary service effects models. Each model was statistically significant and did not show collinearity. Model 3a had the highest R^2^ (42.1%) and the largest explanatory validity.

First, the independent variable in Model 1 was the total number of GPs. The results showed that the total number of GPs had a significant positive relationship with the health management level, which is consistent with Hypothesis 1, which confirms that the higher the medical level is, the higher the primary care service effects are. The R^2^ of the three models were 41.2%, 33.5%, and 25.1%, respectively. Among them, the interpretation of hypertension management was also the highest and had the highest coefficient (8.016), indicating that each additional GP allowed the management of about eight additional hypertension patients. Moreover, the results also indicate that when both times of primary care centers (public and private) have the same level of GPs, the public primary care centers have better management effects.

Secondly, the independent variable in Model 2 was the number of deputy chief physicians, which showed a significant positive relationship with health management levels, verifying Hypothesis 2, which states that the more talent training is developed, the higher the primary care service effects are. Model 2a had the most powerful interpretation, with an R^2^ of 38.4%. The coefficient of Model 2c (28.13) was higher than those of Model 2a and Model 2b, indicating that each additional deputy chief physician allowed for the management of around 28 additional elderly patients. Moreover, the model also indicated that when both types of primary care centers (public and private) have the same level of deputy chief physicians, the public primary care centers have better management effects.

The independent variable in Model 3 was the total number of GPs p and deputy chief physicians. This model passed the significance test, and the R^2^ of Model 3a was the highest, presenting a validity value of 42.1%. Additionally, the total number of GPs and deputy chief physicians was significantly positively correlated with health management, similar to Model 1. Model 3a had the highest coefficient for the total number of doctors, and Model 3c had the highest coefficient for number of deputy chief physicians. The conclusions are similar to Models 1 and 2, representing the stability of the results. Therefore, Models 1, 2 and 3 all showed significantly positive correlations for both types of hospitals (public and private) in the Bao’an and Pingshan districts and indicated that public hospitals had higher health management numbers than private hospitals.

Thirdly, the outpatient costs showed a positive correlation in all three models, verifying Hypothesis 3, which stated that the higher the service levels are, the higher the primary care service effects are. When the hospital’s patient expenses are higher, then it means that more the hospital is managing the health of more patients. However, the total hospital income (financial appropriations plus the hospital’s own income) did not pass the significance test and was thus not necessarily related to health management.

Finally, there was a significant positive correlation between the two-way referral number and health management, with the exception of elderly management in Model 1c and Model 3c, basically verifying Hypothesis 4, indicating that the higher the information sharing level is, the higher the primary care service effects are. The number of two-way referrals indicates the effective implementation of informatization sharing and interaction and cooperation between primary care centers and hospitals, reflecting the influence of healthcare reforms and policy, especially in the management of hypertension and diabetes patients.

In order to further verify the reliability of the research results of the models, a robustness test was carried out. In detail, the interaction items of hospital type and two-way referral number were added, both of which were included in the three models. Table 9 shows the detailed results of the robustness test. The total number of GPs, the number of deputy chief physicians, outpatient expenses, and the interaction terms of the hospital type and two-way referral number all passed the significance test at the level of 0.01. Therefore, the four research hypotheses of this study have been verified, indicating that the research results are robust.

## 5. Conclusions

The government has often adjusted many aspects of rules and policies to promote health equity at the primary level. Although many studies have evaluated healthcare policies in China from the perspectives of the supply of medical and healthcare resources, the utilization efficiency, or the resource allocation degree, some other studies have examined the main factors influencing the effects of the healthcare policies, such as the number of doctors and therapists, support clinics, and home-visit treatments, and little attention has been paid to the interactive process between health care policy formulation and health care implementation and its effects. Thus, policy makers and healthcare workers might have a lack of comprehensive understanding of the problems related to healthcare policies or reforms. Therefore, there are still some problems in current healthcare policy, such as uneven resource distribution, a lack of talent management, etc. [46].

This study reveals that in the process of promoting healthcare reform at the primary level in Shenzhen, the government emphasized the medical and service levels at primary care centers but paid little attention to talent development (especially in terms of deputy chief physicians) and information sharing. However, in the current information age, the use of data has become essential for decision-making in public health at the local level [47], and the development of talent and information sharing play significant roles in promoting medical and health management at the primary level. Therefore, it is suggested that policy formulation and implementation consistency must be emphasized and that policy promotion and influence mechanisms need to be improved to promote the development of medical and health equity effectively at the primary level.

Meanwhile, in the discussion on improving medical and health management at the primary level, most scholars have mentioned the necessity of primary care centers to improve their talent teams and the training of their general practitioners [48]; the level of a medical center largely depends on its general practitioners and medical talents. Through empirical analysis, this study found that the qualifications of doctors and medical talent are key factors that affect health management, verifying that developing the talent of medical teams can greatly improve the service levels of primary care levels [49]. Moreover, this study also found that the service abilities of a primary care center’s deputy chief physicians have a significant impact on the center’s service effects. Therefore, more attention must be paid to developing medical talent when promoting health equity in primary care centers. However, there is still a lack of attention to talent development during policy formulation, with general practitioners having received much more attention than deputy chief physicians. Thus, this study highlights the necessity of highlighting and strengthening talent development in policy formulation and policy implementation, so more medical talents can work at primary care centers to improve the medical services and the overall medical quality and health equity at the primary level.

Additionally, promoting two-way referral between primary care centers and hospitals is an innovative model for integrating and optimizing medical resources at the local level. An active two-way referral helps to promote the flow of high-quality medical resources at the primary level and to improve medical service capacity [50]. This study finds that developing a two-way referral in medical services significantly impacts health management. Two-way referrals are an important embodiment of local medical resource sharing, is conducive to large-scale health network development, strengthens resource allocation, and improves the breadth and depth of medical health equity. However, at present, policy makers pay little attention to medical resources information sharing and the development of two-way referrals, which may lead to an incomplete two-way referral policy and undefined practice protocols, raising information-sharing obstacles between general hospitals and community healthcare service centers [51]. Therefore, policy making in healthcare reform needs to emphasize medical information sharing measures at the primary level, improve homogeneity between policy making and policy implementation, enhance training quality for primary healthcare physicians, and strengthen coordination between primary healthcare institutions and hospitals [52] to enhance policy impact and form a scientific model for health equity at the primary level.

This study may help to provide some insight to increase the effectiveness of healthcare policies. However, due to the limited data, it is difficult to examine broader areas or varied types of primary care centers. Thus, more research about healthcare policies or reforms at the primary level in different regions or countries can be deepened to provide systematic implications to improve the effectiveness of health equity at the primary level.

## Figures and Tables

**Table 1:** Selective or theoretical coding results.

Dimension	Category	Code
Primary Care Service Effects	Management number of hypertension patients	Number of hypertension patients managed
	Management number of diabetic patients	Number of health management of diabetes patients
	Management number of the elderly	Number of elderly health management
Service Levels	Performance appraisal	Compensation Evaluation mechanism
	Medical revenue	Annual outpatient expenses, Annual gross medical income
	Infrastructure	Debt resolving, Infrastructure Construction, Telemedicine Construction
	Pharmaceuticals management	Pharmaceuticals price quality management, pharmaceuticals regulatory, pharmaceutical consumables, drug circulation, the catalogue of medicine
Medical Level	Number of GP practitioners	Establishment general practitioners’ management, local rural talent, general practitioners, the establishment of a general practitioner training mechanism, general practitioner management, general practitioner ad hoc posts, general practitioner titles
	Medical education	Health education, medical teaching coordination, enrollment structure
Talent Level	Deputy Chief Physician of the Center	Innovative scientific research management mechanism, coordinate the allocation of high-level personnel training funds
Information Sharing	Information technology	Internet medical care, Two-way referral

Source: By the authors according to the results of NVivo coding of the official policy documents about healthcare reforms in terms of economic support, basic medical insurance, and internet hospital construction carried out at the District/Municipality level of Shenzhen and Guangdong Province from 2017–2019.

**Table 2:** Dimensions and occupancies.

Dimensions	Primary Care Service Effects	Service Levels	Medical Level	Talent Level	Information Sharing	Total
Frequency	75	71	33	5	8	192
Proportion	39.06%	36.98%	17.18%	2.61%	4.17%	100.00%

Source: By authors. Calculated according to NVivo word frequency.

**Table 3:** Empirical index selection according to analyzed dimensions.

Dimensions	Indexes
Medical Level	Number of GP practitioners
Talent Level	Deputy Chief Physician of the Center
Service Levels	Annual outpatient expenses and annual gross medical income
Information Sharing	Two-way referral
Primary Care Service Effects	Management number of hypertension patients Management number of diabetic patients Management number of the elderly

Source: By authors. Main dimensions from NVivo word frequency and the corresponding indexes for empirical research.

**Table 4:** Variables Setting.

	Variables	Indicators	Index Description
Dependent Variables	Primary Care Service Effects	Management number of hypertension patients	Number of hypertension patients managed as specification
		Management number of diabetic patients	Number of health management of diabetes patients as required
		Management number of the elderly	Number of elderly health management according to specifications
Independent Variables	Medical Level	Number of GP practitioners	Number of GP practitioners
	Talent Level	Number of Deputy Chief Physician of the Center	Number of Deputy Chief Physician of the Center
Control variables	Service Levels	Annual outpatient expenses	
		Annual gross medical income	Including the fiscal revenue
	Information Sharing	Annual number of two-way referrals	Number of Trans cases

**Table 5:** Basic information of the primary care service centers.

Variable Type	Variable Name	Observation Value	Mean	Standard Difference	Minimum Value	Maximum Value
Primary Care Service Effects	Hypertension standard management number	336	290.58	228.62	0.00	1361.00
	Diabetes Management	339	94.55	87.92	0.00	477.00
	Standardized management number of the elderly	338	218.35	199.31	0.00	1220.00
Medical Level	Number of GP practitioners	348	11.08	7.38	0.00	44.00
Talent Level	Number of doctors, deputy chief physician of the center	346	2.52	2.53	0.00	15.00
Service Levels	Outpatient expenses	338	318,182.90	228,592.80	0.00	983,300.00
	Total medical income	338	407,174.30	249,310.30	0.00	991,972.00
	Number of outpatients	338	50,384.45	37,408.76	4.00	226,443.00
Information Sharing	Number of bidirectional referrals	319	4092.84	4677.66	1.00	44,310.00

Source: By the authors. Data for the primary centers in both the Bao’an District and Pingshan District were provided by Shenzhen Municipal Health Commission from 2018–2019.

**Table 6:** Basic information of primary care Service centers in different districts.

Area	Pingshan District				Bao’an District			
	Mean	Maximum Value	Minimum Value	N	Mean	Maximum Value	Minimum Value	N
Number of general practitioners	13.31	44.00	2.00	70	10.52	40.00	0.00	278
Number of deputy chief physician of the center	1.43	8.00	0.00	70	2.80	15.00	0.00	276
Outpatient expenses	285,159.70	983,300	300.39	67	326,347.40	974,599.00	0.00	271
Total medical income	357,312.30	989,180	449.75	67	419,501.80	991,972.00	0.00	271
Number of two-way referrals	3032.18	14,255.00	1.00	61	4343.61	44,310.00	1.00	258
Management number of hypertension patients	157.26	582.00	6.00	65	322.56	1361.00	0.00	271
Management number of diabetic patients	49.54	206.00	0.00	65	105.23	477.00	0.00	274
Management number of the elderly	115.72	412.00	0.00	65	242.78	1220.00	0.00	273

**Table 7:** Correlation analysis.

	Total Number of GP	Deputy Chief Physician Number	Hospital Type	Outpatient Expenses	Hospital Gross Income	Two-Way Referral Number
Total number of GP	1.00					
Deputy chief physician number	0.32	1.00				
Hospital type	−0.16	0.23	1.00			
Outpatient expenses	0.29	0.27	0.18	1.00		
Hospital gross income	0.09	-0.04	0.14	0.24	1.00	
Two-way referral number	0.31	0.16	0.11	0.25	0.08	1.00

**Table 8:** Regression analysis results for Model 1–Model 3.

Variable	Model 1			Model 2			Model 3		
	Model 1a	Model 1b	Model 1c	Model 2a	Model 2b	Model 2c	Model 3a	Model 3b	Model 3c
	Hypertension Patients’ Management	Diabetes Patients’ Management	Elderly Management	Hypertension Patients’ Management	Diabetes Patients’ Management	Elderly Management	Hypertension Patients’ Management	Diabetes Patients’ Management	Elderly Management
Total number of GP	8.02 ***	2.59 ***	7.62 ***				6.92 ***	2.03 ***	4.83 ***
	(1.48)	(0.61)	(1.47)				(1.56)	(0.63)	(1.48)
Deputy chief physician number				15.80 ***	6.67 ***	28.13***	9.47 **	4.81 ***	23.71 ***
				(4.24)	(1.71)	(3.97)	(4.36)	(1.79)	(4.14)
Hospital type	156.50 ***	51.51 ***	133.54 ***	98.75 ***	30.84 ***	62.29 **	138.84 ***	42.59 ***	90.28 ***
	(26.74)	(11.03)	(26.53)	(27.09)	(10.96)	(25.38)	(27.8)	(11.42)	(26.42)
Outpatient expenses	43.47 ***	19.72 ***	56.04 ***	50.21 ***	20.70 ***	52.18 ***	38.87 ***	17.37 ***	44.26***
	(13.61)	(5.62)	(13.50)	(13.88)	(5.62)	(13.01)	(13.72)	(5.64)	(13.04)
Hospital gross income	−3.14	−1.07	−13.61	7.86	3.21	3.20	1.62	1.38	−1.16
	(14.17)	(5.84)	(14.06)	(14.64)	(5.928)	(13.72)	(14.29)	(5.87)	(13.58)
Two-way referral number	0.02 ***	0.01 ***	0.003	0.02 ***	0.007 ***	0.005 **	0.02 ***	0.01 ***	0.003
	(0.002)	(0.001)	(0.002)	(0.002)	(0.0009)	(0.002)	(0.002)	(0.0009)	(0.002)
Constant	−487.65 **	−227.87 **	−504.99 **	−624.00 ***	−268.21 ***	−604.30 ***	−488.74 **	−228.55 ***	−509.87 **
	(215.00)	(88.68)	(213.28)	(218.16)	(88.30)	(204.43)	(213.99)	(87.93)	(203.37)
Observations	317	317	317	316	316	316	316	316	316
R^2^	0.412	0.335	0.251	0.384	0.329	0.299	0.421	0.351	0.323

Note: ** and *** show, respectively significant results at the levels of 0.05 and 0.01.

**Table 9:** Results of the robustness test carried out on Model 4–Model 6.

Variable	Model 4			Model 5			Model 6		
	Model 4a	Model 4b	Model 4c	Model 5a	Model 5b	Model 5c	Model 6a	Model 6b	Model 6c
	Hypertension Patients’ Management	Diabetes Patients’ Management	Elderly Management	Hypertension Patients’ Management	Diabetes Patients’ Management	Elderly Management	Hypertension Patients’ Management	Diabetes Patients’ Management	Elderly Management
Total number of GP	6.62 ***	2.13 ***	5.56 ***				5.72 ***	1.70 ***	3.30 **
	(1.40)	(0.57)	(1.41)				(1.43)	(0.59)	(1.38)
Deputy chief physician number				14.93 ***	6.30 ***	28.55 ***	10.72 **	5.04 ***	26.12 ***
				(4.15)	(1.68)	(3.93)	(4.19)	(1.72)	(4.03)
Hospital type × Two-way referral number	0.02 ***	0.008 ***	0.009 ***	0.02 ***	0.008 ***	0.007 ***	0.02 ***	0.007 ***	0.007 ***
	(0.002)	(0.0009)	(0.002)	(0.002)	(0.0008)	(0.002)	(0.002)	(0.0009)	(0.002)
Outpatient expenses	64.17 ***	26.54 ***	68.03 ***	67.41 ***	26.32 ***	57.00 ***	57.38 ***	23.33 ***	51.22 ***
	(13.22)	(5.43)	(13.39)	(13.47)	(5.45)	(12.73)	(13.40)	(5.48)	(12.86)
Hospital gross income	−2.65	−1.04	−9.20	4.49	1.90	3.98	1.86	1.12	2.47
	(14.09)	(5.79)	(14.27)	(14.43)	(5.84)	(13.64)	(14.11)	(5.78)	(13.55)
Constant	−615.15 ***	−268.56 ***	−596.67 ***	−708.92 ***	−294.43 ***	−630.50 ***	−602.41 ***	−262.76 ***	−569.11 ***
	(212.80)	(87.36)	(215.43)	(214.50)	(86.76)	(202.74)	(211.21)	(86.44)	(202.84)
Observations	317	317	317	316	316	316	316	316	316
R^2^	0.412	0.341	0.22	0.395	0.342	0.3	0.424	0.359	0.312

Note: × presents the interaction terms. ** and *** show significant results at the levels of 0.05, and 0.01, respectively.

## Data Availability

The data presented in this study are available on request from the corresponding author.
